# PRIP: A Protein-RNA Interface Predictor Based on Semantics of Sequences

**DOI:** 10.3390/life12020307

**Published:** 2022-02-18

**Authors:** You Li, Jianyi Lyu, Yaoqun Wu, Yuewu Liu, Guohua Huang

**Affiliations:** 1 School of Electrical Engineering, Shaoyang University, Shaoyang 422000, China; youli9609@163.com (Y.L.); ljy990309@163.com (J.L.); 2 Hunan Key Laboratory for Computation and Simulation in Science and Engineering, Xiangtan University, Xiangtan 411105, China; 201731510071@smail.xtu.edu.cn; 3 College of Information and Intelligence, Hunan Agricultural University, Changsha 410128, China; yuewuliu@whu.edu.cn

**Keywords:** RNA-protein interactions, word2vec, xgboost, embedding, semantics

## Abstract

RNA–protein interactions play an indispensable role in many biological processes. Growing evidence has indicated that aberration of the RNA–protein interaction is associated with many serious human diseases. The precise and quick detection of RNA–protein interactions is crucial to finding new functions and to uncovering the mechanism of interactions. Although many methods have been presented to recognize RNA-binding sites, there is much room left for the improvement of predictive accuracy. We present a sequence semantics-based method (called PRIP) for predicting RNA-binding interfaces. The PRIP extracted semantic embedding by pre-training the Word2vec with the corpus. Extreme gradient boosting was employed to train a classifier. The PRIP obtained a SN of 0.73 over the five-fold cross validation and a SN of 0.67 over the independent test, outperforming the state-of-the-art methods. Compared with other methods, this PRIP learned the hidden relations between words in the context. The analysis of the semantics relationship implied that the semantics of some words were specific to RNA-binding interfaces. This method is helpful to explore the mechanism of RNA–protein interactions from a semantics point of view.

## 1. Introduction

Proteins and RNAs are two of the most important biological macromolecules that constitute life. They exert roles in many biological processes, such as protein synthesis, DNA repair, DNA replication, regulation of gene expression, and viral replication, by interacting with each other [1,2,3,4,5]. Increasing evidence shows that aberrant protein-RNA interactions are closely associated with many complex human diseases [5,6,7,8,9,10,11,12,13,14,15,16,17], such as Alzheimer’s disease [12,13,14], tumors [15], lung cancer [16], and cardiovascular diseases [17]. Therefore, precisely identifying protein-RNA interfaces not only helps understand the mechanism of protein-RNA interactions and provides insight into the pathological mechanisms related to diseases, but also contributes to drug discovery and development.

It is a challenging task to quickly and accurately identify the RNA-binding interface [18,19]. The current experimental methods are capable of accurately detecting RNA-binding sites but are very costly and time-consuming. On the other hand, the computational methods are able to screen protein-RNA interfaces inexpensively and on a large scale, but their accuracy is discouraging. In spite of this, computational methods can inform experimental methods in finding potential RNA-binding interfaces. Over the past decades, many computational methods have been developed for predicting RNA-binding interfaces or RNA–protein interactions [18,19,20,21,22,23,24,25,26,27,28,29,30,31,32,33,34,35,36,37,38,39,40,41,42,43,44]. These computational methods fall into the framework of machine learning, which has two major components: features and algorithms. According to the used features, these computational methods are grouped into three categories: sequence-based, structure-based, and hybrid methods [45]. The sequence-based methods extract informative features directly from primary protein sequences, including widely used position-specific scoring matrices (PSSMs) [40], which are generally computed using PSI-BlAST [46], physicochemical properties of amino acids, and pseudo amino acid composition [47]. Most of the sequence-based methods are easy to understand and compute, but they are insufficient to characterize RNA-binding interfaces. In addition, the computation of PSSMs requires large-scale reference datasets and thus is time-consuming. The structure-based methods extract structural features that are beneficial for improving the prediction of the RNA-binding interface. However, the actual structures of most proteins are not available, and the structures predicted by most computational methods generally contain noise. The hybrid methods inherit the strength of both the sequence-based and the structure-based methods, but they also absorb their shortcomings. Three types of methods also suffer from local interferences [33]. 

Protein sequences are very similar to sentences in the natural language, where each word has a semantic context. The advance in natural language processing (NLP) makes it easy to seize the semantics of words from the context. For example, the Word2vec [48,49] translates words into embedding, making it easy to measure semantic relationships between words. The NLP techniques have been successfully applied to a wide range of areas, including sentiment analysis, spam detection, machine translation, and question answering [50], over the past decades. The NLP techniques have also been recently utilized in the area of bioinformatics [51,52,53,54]. For instance, the long-short term memory (LSTM), a technique of NLP, along with the Word2vec were used for identifying antibacterial peptides in protein sequences [54] and predicting human–virus protein–protein interactions [51]. For more examples, readers can refer to three relevant reviews [55,56,57]. Inspired by the success of NLP, we presented a protein-RNA interface predictor based on the semantics of protein sequences (called PRIP). The PRIP used the Word2vec to extract the semantic embedding of protein sequences and employed the extreme gradient boosting (XGBoost) to discriminate between RNA-binding interfaces and non-interfaces.

## 2. Materials and Methods

### 2.1. Datasets

For a fair comparison with the state-of-the-art methods, we used the same datasets as aPRBind [20]. Namely, the RB198 [22] was used as the training set and the RB111 [40] as the independent set; both were downloaded from http://ailab-projects2.ist.psu.edu/RNABindRPlus/data.html (Accessed on 13 January 2021). The RB198 compiled by Lewis et al. [58] contains unique 198 protein chains. The RB111 is a recently compiled dataset of RNA-binding protein complexes, consisting of 111 protein chains. In both the RB198 and the RB111, the intra-sequence identities are less than 0.3. The chains in the RB111 have less than 0.4 sequence identity with those in the RB198 and the RB44 constructed by Puton et al. [59]. This is a sufficient reason to use the RB111 as the independent set. 

A residue with at least one atom closer than 5 Å to any atoms of RNAs was referred to as the interface residue [20]. According to the definition, the RB198 contains 7950 interface residues and 45,710 non-interface residues, and the RB111 has 3305 interface residues and 34,255 non-interface residues.

### 2.2. Methodology

**Figure 1:**
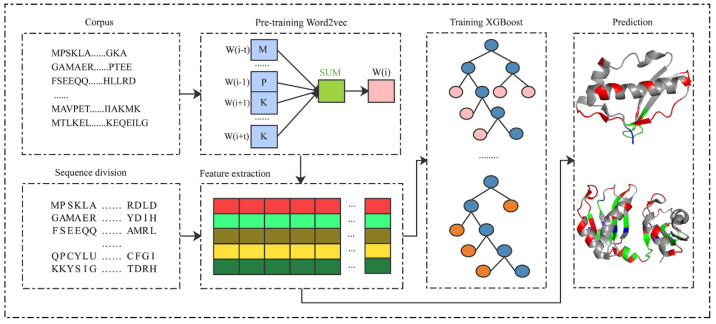
The framework of the PRIP.

As shown in Figure 1, the proposed PRIP consisted of five steps: pre-training the Word2vec [48,49], splitting protein sequences, extracting semantic features, training the XGBoost classifier, and distinguishing between binding and non-binding sites. The corpus of protein sequences was first collected to pre-train the Word2vec. Then, the protein sequences were divided into segments of fixed length. Next, segments were mapped into the semantic features by the pre-trained Word2vec. The semantic features of the training set were used to train an XGBoost classifier. Finally, the trained XGBoost classifier discriminated the binding from non-binding sites, given the inputs of semantic features of segments.

#### 2.2.1. Word2vec

Word2vec, proposed by Mikolov et al. [48,49], is a popular algorithm for embedding representations of words. In fact, Word2vec is a general model of neural network that consists of the input, the hidden, and the output layer. The input consists of one-hot encoding vectors, and the theoretical output consists of one-hot representations of words. The input is mapped into the output by multiplying the linking weights between the input and the hidden layer, and then multiplying the linking weights between the hidden and the output layer. The goal of Word2vec is to minimize residues between the theoretical and the actual output. Word2vec has two computational structures: continuous bag-of-word (CBOW) and skip-gram. The CBOW predicts a target word given a context, while the skip-gram does the opposite, namely predict its context given a target word. For each structure, there are two methods of optimization: hierarchical soft-max and negative sampling. When Mikolov et al. [48,49] applied Word2vec to analyze analogical reasoning tasks, some underlying semantic relationships between words were uncovered. An interesting example is that vec(“Russia”) + vec(“river”) is close to vec(“Volga River”), and vec(“Germany”) + vec(“capital”) is close to vec(“Berlin”). Due to its efficiency and effectiveness, Word2vec is increasingly attracting attention from the natural language processing community. For more details about Word2vec, readers can refer to the relevant reports [60,61]. Here, we adopted for calculation the Gensim [62], a python tool for the Word2vec algorithm. The Gensim is an open source toolkit available at https://radimrehurek.com/gensim/# (Accessed on 5 March 2021). The parameters of the Word2vec are shown in Table 1.

#### 2.2.2. Sequence Division

Protein sequences differ in length. Some are long, while some are short. The protein sequences of variable length are disadvantageous to be subsequently processed by the machine learning algorithms, because the latter generally requires the input to be length-fixed. Therefore, the primary protein sequences must be divided into length-fixed segments. For each residue in the protein sequence, a length-fixed segment was separated from it. The cut residue was located at the center of the segment, and n residues were located downstream and upstream of the segment. At the start or end of the sequence, the corresponding number of X was added to the segment. For example, a protein sequence was assumed to be TGDFPLO, with n as 3. The protein sequence was split into XXXTGDF, XXTGDFP, XTGDFPL, TGDFPLO, GDFPLOX, DFPLOXX, and FPLOXXX. The segments with the interface residue at the center were positive examples in the training set and the independent set and were otherwise considered negative. 

#### 2.2.3. Feature Extraction

We used the 198 protein sequences as the corpus to pre-train Word2vec, where each amino acid was viewed as a word. The pre-trained Word2vec was like a semantic dictionary, where each word (amino acid) corresponded to a semantic vector. Using the semantic dictionary, each residue in the segment was mapped into a semantic vector. Concatenating all the semantic vectors in the segment made it possible to obtain the semantic features of the segment.

#### 2.2.4. XGBoost 

XGBoost, proposed by Chen et al. [63], is an improved GBDT (Gradient Boosting Decision tree) algorithm. The XGBoost has the advantages of high efficiency, flexibility, and portability over the traditional GBDT. Similar to the random forest, the XGBoost is an ensemble learning algorithm. The XGBoost generally consists of many decision trees. The outputs of all decision trees are combined as the final output of the XGBoost. Unlike the random forest, the XGBoost is an additive model, where a new decision tree is fitted by the residues between the actual and the sum of all the previous trees.

Given a training set, $D=\{\left(x_{i},y_{i}\right)|x_{i}\in R^{m},y_{i}\in R\}$, where *n* and *m* denote the number of samples and the dimensions of features, respectively. The XGBoost was assumed to consist of K functions (also called classification or regression trees), namely $F=\left\{f^{1}\left(x\right),f^{2}\left(x\right),\ldots ,f^{K}\left(x\right)\right\}$. The predictive output ${\hat{y}}_{i}^{K}$ for the sample $x_{i}$ is the sum of the output values of all the functions $f^{k}$ (*k* = 1, 2, 3, ……, *K*), namely

(1) $${\hat{y}}_{i}^{K}=\sum_{k=1}^{K}f^{k}\left(x_{i}\right),$$

where $f^{k}\left(x_{i}\right)$ denotes the predictive score of the *k*-th tree.

Assume that the previous t-1 trees are known. The goal of the XGBoost is to look for the t-th tree so as to minimize the sum of the loss between the predictive and the target output. The objective of the XGBoost is modeled as

(2) $$\mathrm{obj}=\sum_{i=1}^{n}l(y_{i}{\hat{,y}}_{i}^{t}),$$

where $y_{i}$ is the target for the sample $x_{i}$, and ${\hat{y}}_{i}^{t}$ is the predictive output of all the *t* trees, which is computed by

(3) $${\hat{y}}_{i}^{t}={\hat{y}}_{i}^{t-1}+f^{t}\left(x_{i}\right),\;$$

The function *l* denotes the loss function, which measures residues between the predictive output ${\hat{y}}_{i}^{t}$ and the target $y_{i}$. In order to reduce or remove over-fitting, regularization is employed. The objective with the regularization is expressed as

(4) $$\mathrm{obj}=\sum_{i=1}^{n}l(y_{i}{\hat{,y}}_{i}^{t})+\sum_{i=1}^{t}\Omega \left(f^{t}\right)\;\;=\sum_{i=1}^{n}l(y_{i}{\hat{,y}}_{i}^{t-1}+f^{t}\left(x_{i}\right))+\Omega \left(f^{t}\right)+\mathrm{constant},$$

where

(5) $$\Omega \left(f_{t}\right)=\gamma T+\frac{1}{2}\lambda \sum_{j=1}^{T}\omega_{j}^{2}\;$$

In Equation (5), λ and γ are two user-defined hyper-parameters, T is the number of leaf nodes, and $\omega_{j}$ is the weight of the *j*-th leaf node. Different from the traditional GBDT, which uses the first-order Taylors, the XGBoost [63] uses the second-order Taylors to approximate the loss function, namely

(6) $$l\left(y_{i},{\hat{y}}_{i}^{t-1}+f_{t}\left(x_{i}\right)\right)\approx l\left(y_{i},{\hat{y}}_{i}^{t-1}\right)+g_{i}f^{t}\left(x_{i}\right)+\frac{1}{2}h_{i}{\left[f^{k}\left(x_{i}\right)\right]}^{2}$$

where $g_{i}$ is the first-order derivative of the loss function,

(7) $$g_{i}=\frac{\partial l\left(y_{I},{\hat{y}}_{i}^{\left(t-1\right)}\right)}{\partial {\hat{y}}_{i}^{\left(t-1\right)}}$$

And $h_{i}$ is the second-order derivative of the loss function,

(8) $$h_{i}=\frac{\partial^{2}l\left(y_{i},{\hat{y}}_{i}^{\left(t-1\right)}\right)}{\partial {\hat{y}}_{i}^{\left(t-1\right)}\partial {\hat{y}}_{i}^{\left(t-1\right)}}$$

Because the constant is not influential on the derivative, the objective is equivalently expressed as

(9) $$\mathrm{obj}=\sum_{i=1}^{n}\left[g_{i}f^{t}\left(x_{i}\right)+\frac{1}{2}h_{i}{\left[f^{t}f_{t}^{2}\left(x_{i}\right)\right]}^{2}\right]+\gamma T+\frac{1}{2}\lambda \sum_{j=1}^{T}\omega_{j}^{2}\;$$

Let $I_{j}$ be the set of samples belonging to the *j*-th leaf node, namely

(10) $$I_{j}=\left\{i|q\left(x_{i}\right)=j\right\}\;$$

where $q\left(x_{i}\right)$ represents the structure of the *t*-th decision tree. Let

(11) $$G_{j}=\sum_{i\in I_{j}}^{⬚}g_{i}$$

Be the sum of the first-order derivative over all the samples of the *j*-th leaf node, and

(12) $$H_{j}=\sum_{i\in I_{j}}^{⬚}h_{i}\;$$

Be the sum of the second-order derivatives over all the samples of the *j*-th leaf node. The objective is further simplified as

(13) $$\mathrm{obj}=\sum_{j=1}^{T}\{G_{j}\omega_{j}+\frac{1}{2}\left(\lambda +H_{j}\right)\omega_{j}^{2}\}+\gamma T$$

Equation (13) is univariate and quadratic. If the structure of the decision tree was fixed, the objective could have the minimum. If, and only if, the weight of the leaf node was set to

(14) $$\omega_{j}^{*}=-\frac{G_{j}}{H_{j}+\lambda }$$

The minimum of the objective was computed by

(15) $${\mathrm{obj}}^{*}=-\frac{1}{2}\sum_{j=1}^{T}\frac{G_{i}^{2}}{H_{i}+\lambda }+\gamma T.$$

The descriptions above introduced how to optimize the weights of the leaf nodes given the fixed structures of trees. It is easy to understand and realize, but the optimization of the tree structure is an NP-complete question. The number of trees would increase exponentially with the increasing number of samples. It is impossible in practice to enumerate all possible trees to reach the global optimum solution. A practical solution is to adopt the greedy algorithm. The XGBoost begins with one leaf node and expands to new branches iteratively. The new expanded tree was assumed to be with the left branch L and the right branch R. The gain of the objective was computed by

(16) $$\mathrm{Gain}=\frac{1}{2}\frac{{\left(\sum_{i\in I_{L}}g_{i}\right)}^{2}}{\sum_{i\in I_{L}}h_{i}+\lambda }+\frac{1}{2}\frac{{\left(\sum_{i\in I_{R}}g_{i}\right)}^{2}}{\sum_{i\in I_{R}}h_{i}+\lambda }-\frac{1}{2}\frac{{\left(\sum_{i\in I}g_{i}\right)}^{2}}{\sum_{i\in I}h_{i}+\lambda }-\gamma$$

The tree with the minimum gain was chose for the next possible expansion. 

### 2.3. Evaluation Metrics

The k-fold cross validation and the independent test are the commonly used ways of examining the performance of the machine learning algorithms. In the k-fold cross validations, all the training samples are divided into k parts. The machine learning algorithm is trained by k-1 parts of the sample and is tested by the remaining part. The process is repeated k times. The sensitivity (SN), accuracy (ACC), specificity (SP), and Matthews correlation coefficient (MCC) are used to evaluate the performance, which were computed by

(17) $$\mathrm{SN}=\frac{TP}{TP+FN}$$

(18) $$\mathrm{SP}=\frac{TN}{TN+FP}\;$$

(19) $$\mathrm{ACC}=\frac{TP+TN}{TP+FP+TN+FN}$$

(20) $$\mathrm{MCC}=\frac{TP\times TN-FP\times FN}{\sqrt{\left(TN+FN\right)\left(TN+FP\right)\left(TP+FN\right)\left(TP+FP\right)}}\;$$

where *TP* and *TN* are the numbers of correctly predicted interfacial residues (binding site) and the numbers of correctly predicted non-interfacial residues (non-binding site), respectively. *FP* and *FN* stand for the number of wrongly predicted interfacial residues and non-interfacial residues, respectively. The receiver operating characteristic (ROC) curve is also employed to visualize performances. The ROC curve draws true positive rates (SN) against false positive rates (1-SP) under various thresholds. The area under ROC curves (AUROC) is used to quantitatively assess the performance.

## 3. Results

### 3.1. Parameter Optimization

**Figure 2:**
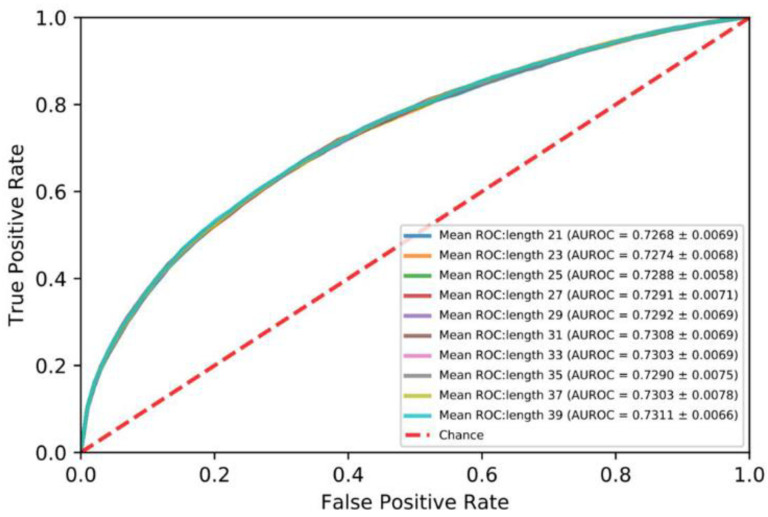
Mean ROC curves for five-fold cross validation: protein sequences 21 to 39.

In order to investigate the impact of the length of the segments on performance, protein sequences were divided into segments ranging from 21 to 39 at an interval of 2. As shown in Figure 2, the AUROC of the segment of 39 amino acid residues is best. Therefore, we set the length of the segment to 39. 

### 3.2. Selection of Models 

**Figure 3:**
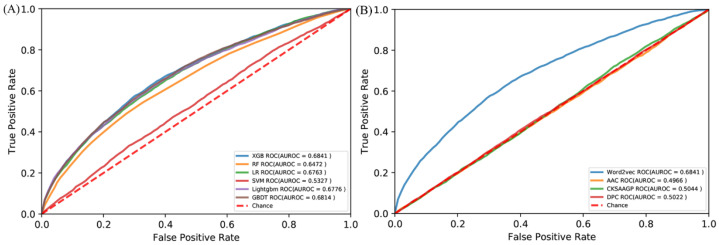
ROC curves of (**A**) different algorithms by the independent test and (**B**) different amino acid encoding methods by the independent test.

There are more than 100 machine learning algorithms that have been applied to a wide range of fields. We compared the XGBoost with five popular algorithms: random forest (RF) [64], support vector machine (SVM) [65], logical regression (LR) [66], gradient boosting decision tree (GBDT) [67], and Lightgbm [68]. All the algorithms were trained by the same RB198 and tested by the identical RB111. The ROC curves are shown in Figure 3A. Obviously, the XGBoost is superior to these five algorithms in terms of predicting RNA-binding protein interfaces. We also compared the word embedding of the Word2vec with three common representations: amino acid composition (AAC) [69], dipeptide composition (DPC) [70], and the composition of k-spaced amino acid group pairs (CKSAAPGP) [71]. The AAC calculates the occurring frequency of each amino acid in a given protein or peptide sequence, resulting in a 20-dimensional vector. The DPC calculates the frequency of amino acid pair occurrence, so it is a 20 × 20 = 400 dimensional vector. The CKSAAPGP computes the frequency of amino acid group pairs separated by K amino acids. Here, K was set to 3, and the five groups were the aliphatic group (G, A, V, L, M, I), aromatic group (F, Y, W), positive charge group (K, R, H), negative charge group (D, E), and uncharged group (S, T, C, P, N, Q) [72], so the dimension of the CKSAAPGP is 5^2^ × 4 = 100. The ROC curves are shown in Figure 3B. The embedding of the Word2vec is superior to the three representations. Therefore, we chose semantic embedding of the Word2vec as the representations of proteins and XGBoost as the learning algorithm. 

### 3.3. Comparison with State-of-the-Art Methods

**Figure 4:**
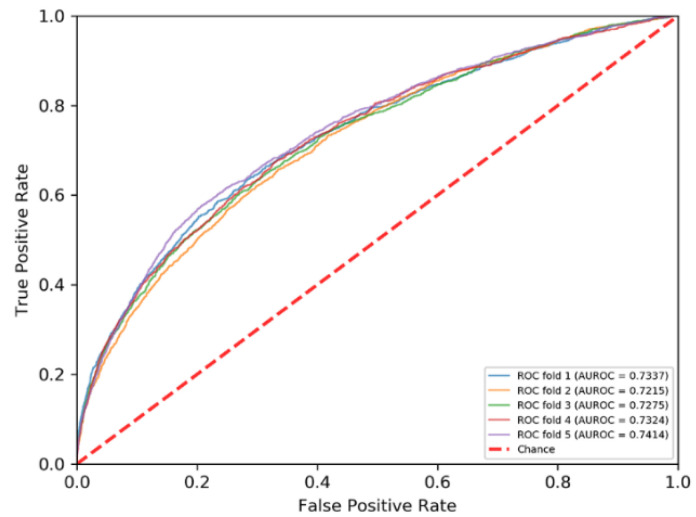
ROC curves of five-fold cross validation.

The PRIP obtained a mean AUROC of 0.73 over five-fold cross validations and an AUROC of 0.68 over the independent test, as shown in Figure 3 and Figure 4. Recently, some methods have been developed for predicting the RNA–protein interface, including aPRBind [20], FastRNABindR [21], RNABindR v2 [22], BindN+ [33], and PPRInt [28]. The aPRBind [20] is a convolutional neural network-based method that uses sequence and structure information, while FastRNABindR [21], RNABindR v2 [22], BindN+ [33], and PPRInt [28] all adopt sequence-based features for interface prediction [20,21,22,28,33]. The performance of the independent test over the RB111 are listed in Table 2. The PRIP increased SN by 0.19 over the aPRBind [20], by 0.06 over the FastRNABindR [21], by 0.04 over the RNABindR v2 [22], by 0.24 over the BindN+ [33], and by 0.19 over the PPRInt. On the other hand, the PRIP performed worst in terms of SP, ACC, and MCC. Apart from the PRIP, the best SN was 0.63, which was obtained by the RNABindR v2 [22]. This implied that it was challenging to correctly predict RNA–protein interfaces. Our method obtained a SN of 0.67. Table 3 lists the performance of the five-fold cross validations over the RB198. The same phenomenon was observed as in the independent test over the RB111. The PRIP obtained a better SN than the aPRBind [20].

### 3.4. Analysis of Pattern of the RNA-Binding Interfaces

**Figure 5:**
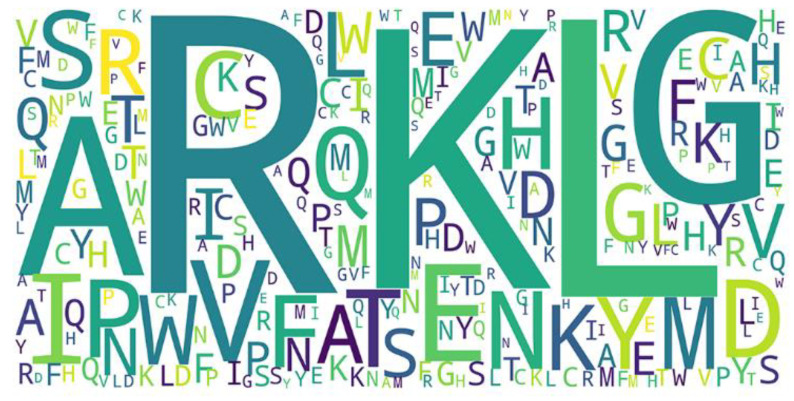
Word cloud of positive samples.

**Figure 6:**
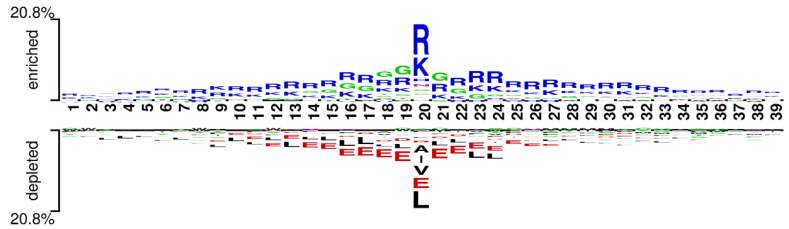
Two Sample logos: positive and negative samples correspond to the upper and lower parts respectively, and the height of residual letters is positively correlated with stacking order and frequency.

We used the word cloud generator to draw a word cloud diagram of positive samples. As shown in Figure 5, the characters R, L, K, and G are dominant in the positive samples. We further employed Two Sample Logo [73] to visualize the difference between RNA-binding and non-binding protein sequences. Two Sample Logo is a tool to calculate and visualize differences between two sets of aligned samples of amino acids. Due to its simplicity and effectiveness, Two Sample Logo has widely been applied to the analysis of sequence patterns, such as post-translational modification patterns [74,75,76]. As shown in Figure 6, the characters R, K, and G are enriched in the RNA-binding protein sequences, and the characters L, A, E, and V are depleted. The results are in agreement with the word cloud diagram (Figure 5). This might imply that RNA-binding interfaces were associated with the emergence of R, K and G. 

## 4. Discussion

We used only semantic embedding of protein sequences generated by the Word2vec to predict RNA-binding interfaces, and obtained competing performances with the state-of-the-art methods, including aPRBind [20], FastRNABindR [21], RNABindR v2 [22], BindN+ [33], and PPRInt [28]. This demonstrated that RNA-binding protein sequences were of semantics. The semantics of protein sequences have recently attracted attention from the molecular biology and bioinformatics communities [77]. For example, semantics were applied to detect remote evolutionary relationships [78,79], to predict protein subcellular localization [80], and to recognize protein–protein interactions [81]. Like natural language, biological sequences formed stable semantic relationships during the course of evolution. This is one of the reasons that our method obtained better performance in predicting RNA-binding interfaces. 

**Figure 7:**
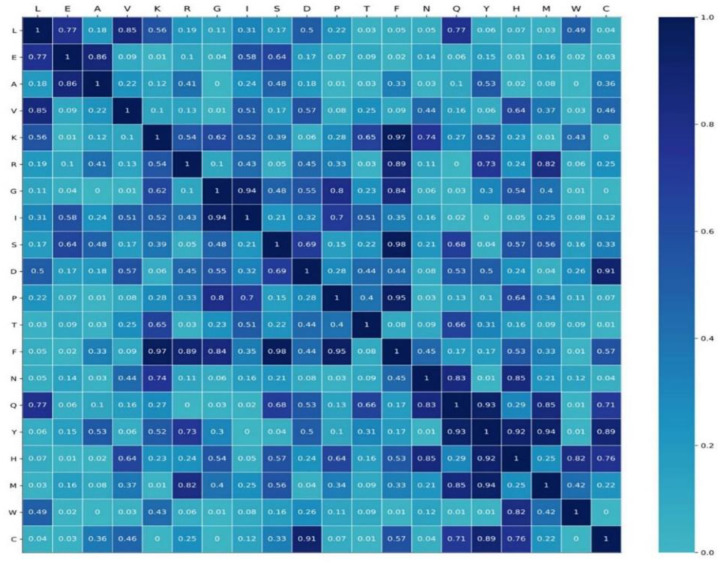
The *p*-value matrix by student’s *t*-test.

In order to investigate the specificity of semantics, we generated four datasets of protein sequences by randomly altering 40%, 45%, 50%, and 55% of residues of RNA-binding protein sequences in the RB198. Each shuffled dataset was used as a corpus to pre-train Word2vec. The semantic relationship between words was defined as the cosine between the embedding of words, namely

(21) $$\cos\theta =\frac{\sum_{i=1}^{n}\left(A_{i}\times B_{i}\right)}{\sqrt{\sum_{i=1}^{n}{\left(A_{i}\right)}^{2}\times }\sqrt{\sum_{i=1}^{n}{\left(B_{i}\right)}^{2}}}\;$$

where $\left(A_{1},A_{2},\cdots ,A_{n}\right)$ and $\left(B_{1},B_{2},\cdots ,B_{n}\right)$ are the semantic embedding of the word A and B, respectively. Four shuffled datasets generated four stochastic semantic relationships for any two words. We used the RB198 as a corpus to pre-train the Word2vec, and we set the epochs to 100, 200, 300, and 400. We obtained four semantic embeddings of words. Using Equation (21), we computed true semantic relationships between any two words. We used student’s test to investigate the difference in semantic relationships. As shown in Figure 7, some parts of the semantic relationship are not of significant difference, while some are of significant difference (*p*-value < 0.05). For example, for L, the semantic relationship with three amino acids (T, M, and C) is of significant difference, while the semantic relationship of W with up to eight amino acids (E, A, V, G, F, Q, Y, C) is of significant difference. This indicated that some semantic relationships were specific to RNA-binding protein sequences. 

As shown in Table 2 and Table 3, the PRIP didn’t show marked superiority over the state-of-the-art methods. This is because some negative segments are similar to the positive segments. For example, the protein sequence ‘VERIFPL’ has an RNA-binding interface, namely I. The protein sequence was assumed to be divided into three segments of five residues, ‘VERIF’, ‘ERIFP’, and ‘RIFPL’. The interface was located at the center of the second segment, and thus it was a positive sample and the other two were negative ones. In fact, the two negative samples were very similar to the positive one, with only one different amino acid residue. Therefore, these semantics were too close to discriminate. After disrupting the negative fragments of the RNA-binding interface, we retrained the PRIP model (named it PRIP *) and repeated five-fold cross validation and the independent test. Table 4 shows the predictive performance. Obviously, SN and SP both increased, but the increase did not reach the expected value. There might be two reasons. One was that the original semantics of the negative samples were lost if we disrupted the sequence of all the negative samples. The other was that the motif of RNA-binding interfaces was quite complicated.

**Figure 8:**
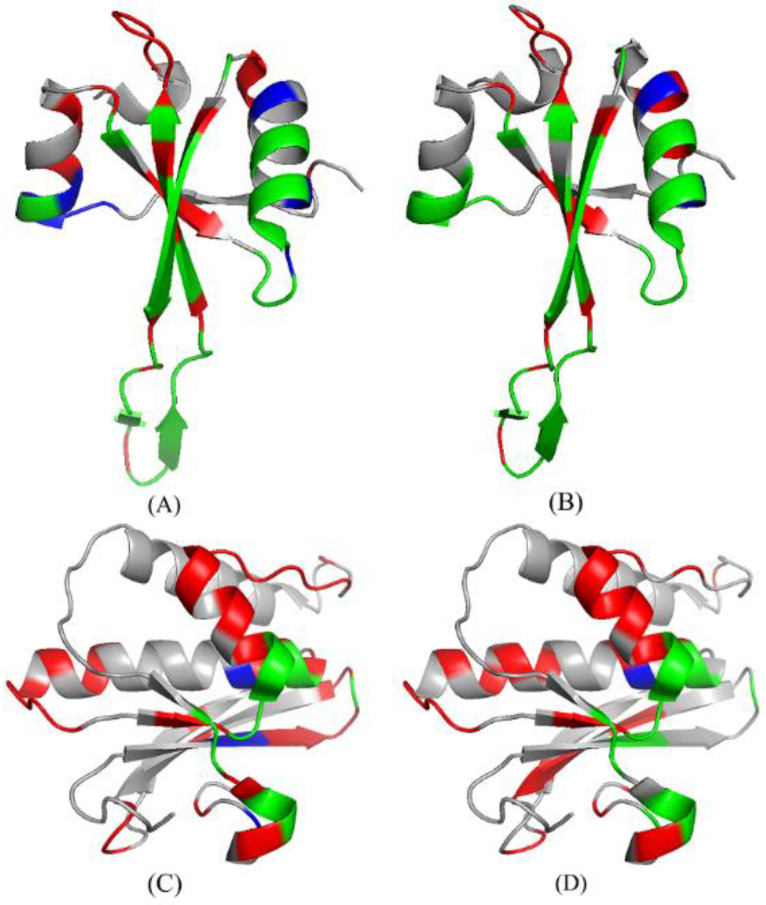
The protein structures of the predicted results of 4V90_56 and 3ULD_ A in PRIP and PRIP *. (**A**,**B**) represent the prediction results of 4V90_56 on PRIP and PRIP*, respectively. (**C**,**D**) represent the prediction results of 3ULD_ A on PRIP and PRIP*, respectively. Green, red, and blue represent TP, FP, and FN, respectively.

As shown in Table 5, we conducted an analysis of two cases: 4V90_56 [82] and 3ULD_ A [83]. The predictive performances are summarized in Table 5 for the RNABindRPlus [40], the PRIP, the PRIP *. Obviously, the PRIP * obtained the best SN, which is 0.16 more than that of the RNABindRPlus [40] and 0.12 more than that of the PRIP over the 4V90_56. Over the 3ULD_ A, the SN of the PRIP * is 0.13 more than that of the RNABindRPlus [40] and 0.20 more than that of the PRIP. Figure 8 illustrates the predicted structure of proteins in the gray cartoon. 

## 5. Conclusions

RNA–protein interactions play key roles in the regulation of many cellular processes and are increasingly becoming a hot topic. Although many computational methods have been presented in the past decades, it is still a challenging task to precisely and cheaply detect RNA-binding interfaces. We presented a sequence semantics-based method to predict RNA-binding interfaces. Compared with the state-of-the-art methods, the presented method learned the hidden relations between words in the context. This method is helpful to explore the mechanism of RNA–protein interactions from a semantics point of view. 

## Figures and Tables

**Table 1:** The set of the parameters in Word2vec.

Name	Values
Structure	CBOW
Vector Size	25
Corpus	RB198
Window size	5
Negative sampling	5
Epoch	200
Workers	1

**Table 2:** PRIP was compared with existing methods on RB111.

Methods	SN	SP	ACC	MCC
PRIP	0.67	0.60	0.61	0.16
aPRBind [20]	0.48	0.90	0.86	0.32
FastRNABindR [21]	0.61	0.76	0.75	0.24
RNABindR v2 [22]	0.63	0.73	0.72	0.22
BindN+ [33]	0.43	0.87	0.84	0.24
PPRInt [28]	0.48	0.79	0.76	0.18

**Table 3:** The average results of the five-fold cross-validation studies on RB198 when compared to aPRBind.

Methods	SN	SP	ACC	MCC
PRIP	0.73	0.60	0.62	0.23
aPRBind [20]	0.65	0.82	0.74	0.48

**Table 4:** Performances by the PRIP and the PRIP *.

	Methods	SN	SP	ACC	MCC
Five-fold cross validation	PRIP	0.73	0.60	0.62	0.23
	PRIP *	0.74	0.63	0.65	0.27
Independent test	PRIP	0.67	0.60	0.61	0.16
	PRIP *	0.69	0.62	0.63	0.18

**Table 5:** The predictive performances over the 4V90_56 and the 3ULD_A.

Protein	Methods	SN	SP	ACC	MCC
4V90_56	RNABindRPlus [40]	0.79	0.82	0.81	0.61
	PRIP	0.83	0.57	0.69	0.41
	PRIP *	0.95	0.65	0.78	0.62
3ULD_ A	RNABindRPlus [40]	0.80	0.87	0.86	0.53
	PRIP	0.73	0.67	0.68	0.26
	PRIP*	0.93	0.71	0.74	0.42

## Data Availability

The data and source code can be found here: https://github.com/Good-Ly/PRIP.git (Accessed on: 4 February 2022).
